# Closing an Intractable Tracheoesophageal Fistula Caused by a Tracheoesophageal Shunt Using a Myocutaneous Flap and a Hinged Flap With Skin Graft in a Two-Step Procedure

**DOI:** 10.7759/cureus.15913

**Published:** 2021-06-25

**Authors:** Yasuyuki Morimatsu, Koichiro Yonezawa, Hidetoshi Matsui, Shigemichi Iwae, Shunsuke Sakakibara

**Affiliations:** 1 Department of Plastic Surgery, Kobe University Hospital, Kobe, JPN; 2 Department of Head and Neck Surgery, Hyogo Cancer Center, Akashi, JPN; 3 Department of Otolaryngology, Nishikawa ENT Clinic, Higashi Osaka, JPN; 4 Department of Plastic Surgery, Kobe University Graduate School of Medicine, Kobe, JPN; 5 Department of Plastic Surgery, Hyogo Cancer Center, Akashi, JPN

**Keywords:** tracheoesophageal fistula, tracheoesophageal shunt, free jejunal reconstruction, high-frequency jet ventilation, prelaminated hinged flap

## Abstract

Total laryngectomy involves removal of the vocal cords resulting in the loss of vocal function. After laryngectomy, the patient's vocal function can be restored in several ways, including the insertion of a tracheoesophageal (TE) shunt. A TE shunt is considered an effective means of restoring speech due to its high efficacy, low requirement for training, and no need for any equipment while speaking. However, complications such as saliva inflow into the trachea, caused by the widening of the shunt opening, have also been reported. Moreover, the optimal treatment for an enlarged fistula has not yet been established.

A fistula may also form at sites of hypopharyngeal reconstruction with free jejunal transplantation. Following its formation, the influx of saliva, infections, and pressure exerted by the act of swallowing make a fistula resistant to closure, and most patients require closure surgery using myocutaneous flaps.

We encountered a case where an intractable TE fistula formed due to a TE shunt after the patient underwent total pharyngolaryngeal resection for hypopharyngeal cancer and hypopharyngeal reconstruction with a free jejunum flap. Since the optimal method for the TE fistula closure remains uncertain, we attempted to close the fistula according to the fistula closure of the free jejunal transplantation.

Failure to close a TE fistula using a myocutaneous flap necessitates a re-closure procedure. However, because the surgical field around the trachea can be limited in such patients, creating an additional myocutaneous flap may not be feasible. In addition to the myocutaneous flap, ventilation control using a conventional intubation tube may further narrow the surgical field during the re-closure surgery. Based on our experience and existing literature, in this article, we summarize several ways of managing TE fistula when the surgical field around the trachea is limited.

## Introduction

Total laryngectomy, which includes removal of the vocal cords, results in loss of vocal function. However, the patient's vocal function can be restored in several ways [1]. Inserting a tracheoesophageal (TE) shunt is a method of voicing. It involves creating a shunt between the residual tracheal mucosa and the esophageal mucosa after laryngectomy. When the tracheal opening is closed during expiration, air flows into the esophagus through the shunt and causes the hypopharyngeal mucosa to vibrate [2]. Voice acquisition with a TE shunt has merit for speech acquisition with a high speech acquisition rate, low need for training, and no requirement of any equipment during speech [3]. However, in addition to complications, such as tracheal stenosis and occlusion of the shunt by granulation tissue that may occur at the permanent tracheal foramen alone, the inflow of saliva into the trachea due to the widening of the opening of the shunt has been identified as a complication [4,5]. This arises because the communication between the tracheal mucosa and the esophageal mucosa is close to the tracheal foramen. The best treatment for an enlarged fistula has not yet been established, and there are many reports of closure methods that range from simple closure to closure with a myocutaneous flap, which has a good blood flow [6].

Fistula formation may also occur at sites of hypopharyngeal reconstruction with free jejunal transplantation [7-10]. Once a fistula is established, the influx of saliva, infections, and pressure due to swallowing makes the fistula resistant to closure. In some cases, spontaneous closure or re-suturing may occur. However, in most cases, closure surgery, especially using myocutaneous flaps, is required [11].

Herein, we report the closure of an intractable TE fistula caused by a TE shunt after total pharyngolaryngeal resection for hypopharyngeal cancer and reconstruction with a free flap of jejunum. The successful closure of the intractable fistula required a two-step procedure using a myocutaneous flap and a hinged flap with a skin graft.

When the closure of a TE fistula with a myocutaneous flap fails, another re-closure is required. However, because the surgical field around the trachea in such patients can be limited, creating an additional myocutaneous flap may not be possible. In addition to the myocutaneous flap, ventilation control using a conventional intubation tube may further narrow the surgical field during the re-closure surgery. In this article, we suggest several ways to manage such cases when there is a limited surgical field around the trachea.

## Case presentation

This is the case of a 66-year-old man with no past medical history.

Current medical history

Total pharyngolaryngeal resection, free jejunal reconstruction, permanent tracheal creation, and TE shunting were performed after a diagnosis of hypopharyngeal carcinoma (cT4aN2bM0) was made. On postoperative day 11, signs of infection, such as erythema around the tracheal foramen that was around the TE shunt, were observed. Consequently, administration of antibiotics commenced the same day, and conservative management, involving debridement and lavage, was performed. However, this resulted in tissue necrosis, which progressed and the fistula extended to the jejunal anastomosis; therefore, a spontaneous closure was deemed unlikely.

Present symptoms

**Figure 1:**
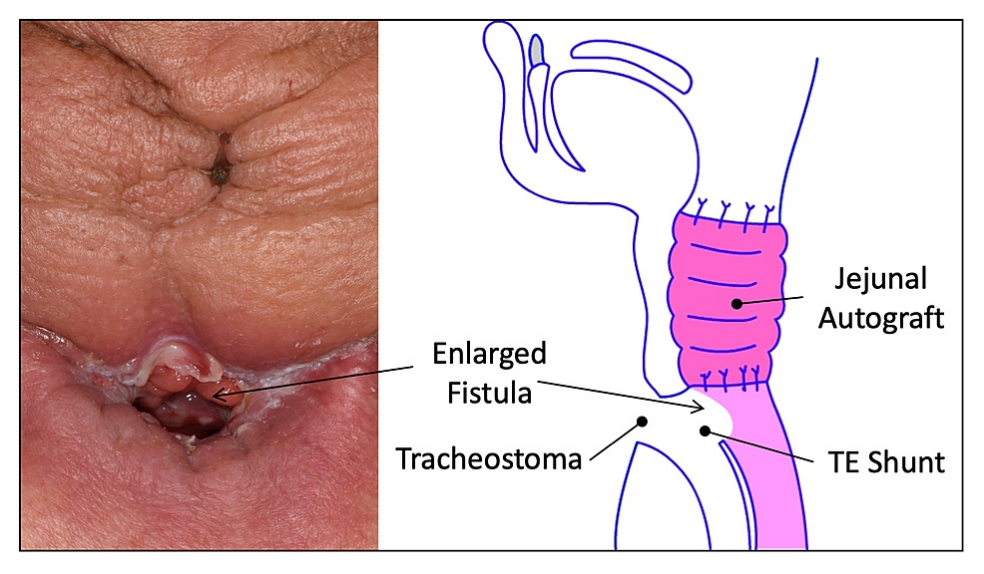
Present symptom of our case (left) and its scheme (right).

Erythema around the permanent tracheal foramen was noticed. The TE shunts enlarged and a tracheoesophageal fistula was seen at the posterior side of the tracheal foramen (Figure 1).

Progress

Preoperative upper gastrointestinal endoscopy (GE) was performed to assess the esophageal mucosa, the jejunal mucosa, and the extent of the fistula. Using GE, the anterior wall of the jejunoesophageal anastomosis was found to be open but the posterior wall was continuous and the blood flow at the edges of the fistula was good. Therefore, it was judged that the anterior wall of the jejunoesophageal anastomosis could be sutured sufficiently, and 18 days after the creation of the TE shunt, closure of the fistula was planned under general anesthesia.

**Figure 2:**
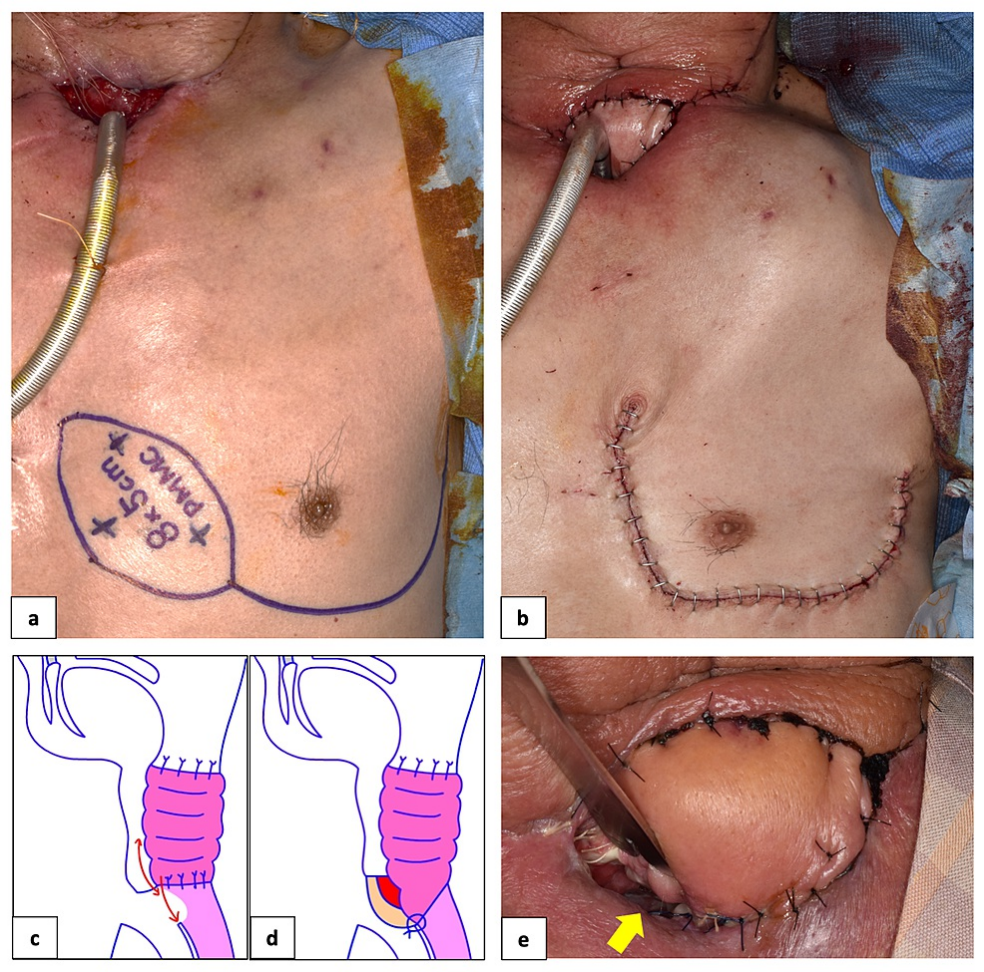
First closure of the fistula and recurrent fistula Closure of the fistula with a pectoralis major myocutaneous flap and each scheme (a-d), and the recurrent fistula 11 days later (e, arrow).

The jejunal flap and its anterior subcutaneous tissue were dissected, and the mucosal and muscular layers of the fistula, along with the extension of the jejunal flap, were sutured according to the Albert-Lembert method (Figure 2c). In addition, a pectoralis major myocutaneous flap measuring 8 × 5 cm, was elevated and secured to the tissue defect on the anterior surface of the jejunal flap (Figures 2a, 2b, 2d), and the tissue with blood flow was used to fill the reattached site. A tracheal cannula was inserted immediately after surgery to prevent tracheal obstruction by flap compression and to maintain the shape of the tracheostomy. After surgery, no signs of infection were observed, but 11 days after the closure procedure, at the pectoralis major myocutaneous flap, a suture opening in the region of the tracheostomy and the jejunoesophageal restart were observed, and the lesion gradually enlarged and re-fistulated (Figure 2e, arrow). This was thought to be because the cannula that was inserted to prevent obstruction of the permanent tracheal hole pressed against the flap and compressed the suture. This led to decreased blood flow and the development of a pressure ulcer, which opened the suture and resulted in a fistula. Endoscopic fistula closure was attempted, but it failed.

**Figure 3:**
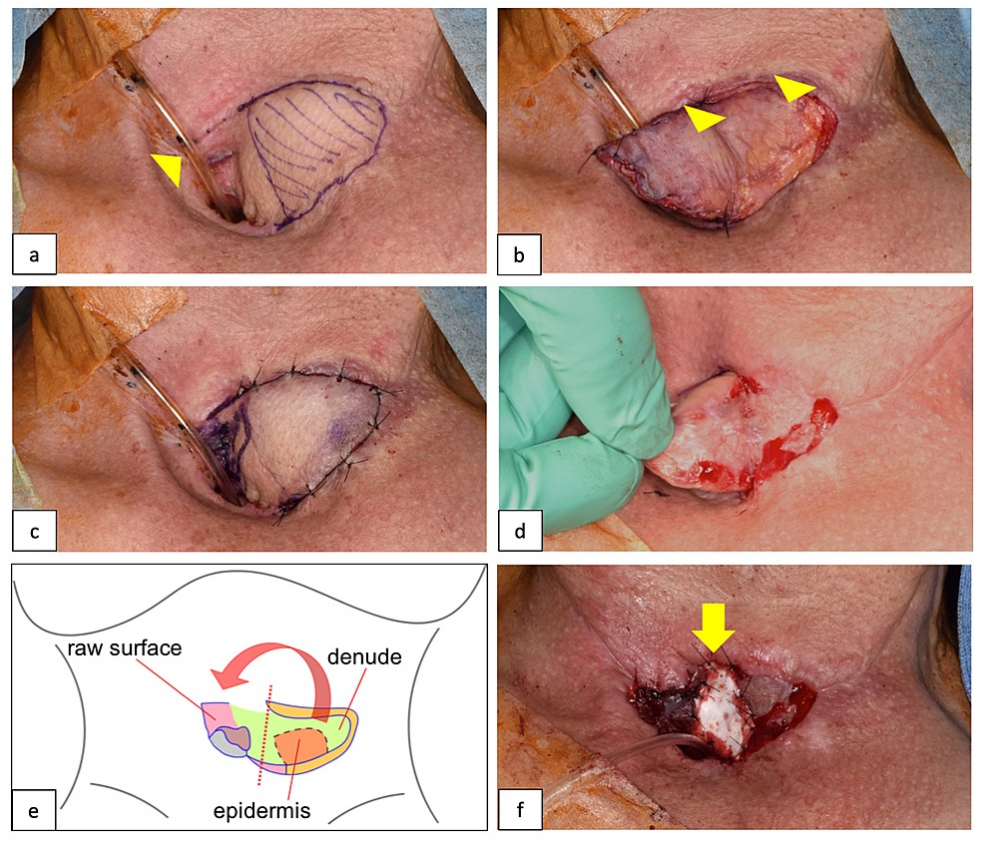
Second re-closure surgeries using a hinged flap Design of the hinged flap and respiratory management using high-frequency jet ventilation (HFJV) (a, arrowhead), Thiersch skin graft on the posterior raw surface of the flap (b, arrowhead), re-elevation of the flap after delay (c,d), an overview of the procedure (e), and an artificial dermis (arrow) on the raw surface created by an additional incision in the center of the skin graft (f, arrow).

Reoperation was planned 19 days after the initial closure. The cervical operative field was occupied by the pectoralis major flap of the previous operation; hence, the working space was further limited. Therefore, the operative field was secured using high-frequency jet ventilation (HFJV) for subsequent intraoperative respiratory management (Figure 3a, arrowhead). During the re-operation, a hinged flap was elevated from the pectoralis major myocutaneous flap that had been transported to the neck during the previous surgery, and the fistula was closed using a two-step procedure. First, under general anesthesia, a part of the major thoracic muscle flap was elevated as a hinged flap, and the flap was delayed using a Thiersch graft from the left femur on a raw surface that appeared on the posterior surface of the flap (Figures 3a-c).

**Figure 4:**
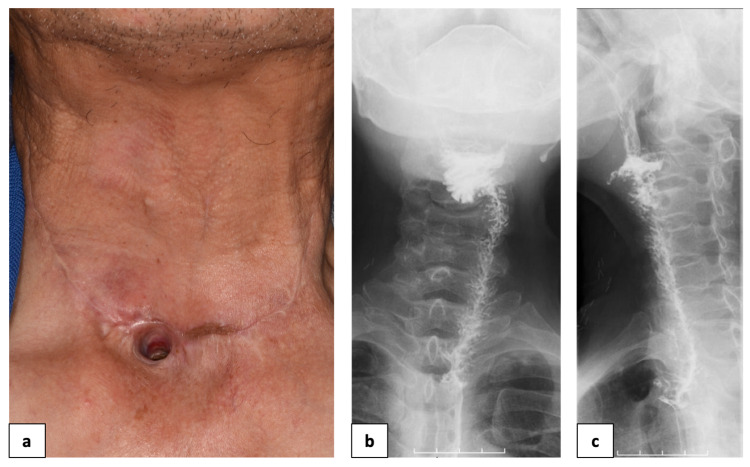
48 days after the last closure No fistula recurrence (a), and swallowing videofluorography (b, c), 48 days after surgery.

A second operation was performed 18 days after flap delay. The fistula was closed by elevating the hinged flap again and inverting it over the fistula. At that time, the anterior part of the hinged flap was denuded except the epidermis for the fistula. The epidermis on the flap was sutured with surrounding tissue of the fistula, and the dermis was attached and sutured with the raw surface area around the fistula so that the epidermis was exposed on the mucosal side when the flap was inverted (Figures 3d, 3e). Furthermore, an additional longitudinal incision was made over the center of the skin graft to sufficiently extend the hinged flap, and the flap was extended (Figure 3f, arrow). The raw surface in the center of the skin graft was incised and covered with an artificial dermis; this healed with scar contractures during the postoperative healing process, and the posterior surface of the tracheal foramen was retracted. About 48 days postoperatively, there was no fistula recurrence, and the permanent tracheal foramen was well-shaped (Figure 4a). A good swallowing function was achieved with no stenosis or leakage at the suture site without stent insertion (Figure 4b).

## Discussion

When total laryngectomy is performed for laryngeal, hypopharyngeal, esophageal, or thyroid cancers, patients lose their vocal function because laryngectomy includes removal of the vocal cords. To salvage this, several measures have been taken to restore the patients’ vocal function. Speech acquisition in patients that undergo laryngectomy can be achieved either through the creation of an esophageal voice, an artificial larynx, or a TE shunt [1]. In this context, the voice produced by the TE shunt is an excellent choice for restoring patients’ vocal function after laryngectomy because of its high acquisition rate and no need for any equipment [2,3]. Therefore, TE shunts are often performed in patients that undergo total laryngectomy.

There are two main methods of shunting: the Amatsu method, which does not use a silicon voice prosthesis, and the other is by mounting of a voice prosthesis on the shunt portion [2,3,12]. TE shunts are associated with fistula formation due to shunt expansion and inflow of saliva into the trachea because a communication is created between the tracheal mucosa and the esophageal mucosa [4,5]. Although a first-line treatment for fistula expansion has not yet been established, the risk of aspiration pneumonitis due to fistula expansion occurs in 39% of patients [13]. Therefore, fistula closure is common, and this is often reportedly performed using local sutures and flaps that have good blood flow [6]. There are few reports of TE shunt fistula closure; therefore, sufficient consideration of adequate closure techniques is needed. However, it is reasonable to decide to close the fistula at the suture site after free jejunal transplantation because the sites of the lesions are similar.

Free jejunal transplantation is the first choice for hypopharyngeal reconstruction after total laryngectomy. This is due to its advantages, such as achievement of better postoperative function, sufficient size for reconstruction, and fewer complications, such as fistula formation, compared to other myocutaneous flaps [7]. However, reports indicate that 3%-32% of cases are associated with dehiscence or fistula formation [7-10]. This is the most common complication of hypopharyngeal reconstruction using free jejunal transplantation after total pharyngolaryngectomy, apart from other common complications, such as postoperative infection. Once a fistula occurs, the influx of saliva, infection and pressure due to swallowing make it difficult to close the fistula.

Detailed reports of fistula closure after free jejunal reconstruction are limited, but Gehrking et al. suggested that only a few patients with localized procedures can be cured by spontaneous closure or re-suturing and that some closure surgery is required in most cases, especially in cases where a myocutaneous flap, such as the pectoralis major flap and the sternocleidomastoid flap, was used [11,14,15]. However, musculocutaneous flaps are large relative to the narrow operative field around the trachea, and it is inevitable that the field of view and working space are limited during surgical manipulation. The tracheal holes are also easily compressed and occluded by flaps; therefore, it is necessary to secure the airway by inserting a cannula postoperatively. In such cases, there is concern about the impact of cannulation on the suture site and the formation of pressure ulcers due to compression of the flap by the cannula.

In this case, the surgical field around the tracheal foramen was infected, and the fistula had extended to the hypopharyngeal-free jejunal suture. Therefore, a muscular flap, such as the pectoralis major flap, was selected to close the fistula after reconstruction of the free jejunum. However, as a result of ventilation control using a conventional intubation tube, the surgical field around the trachea was limited. Therefore, we were unable to obtain sufficient working space while suturing the pectoralis major flap and visibility could not be secured, which made suturing difficult. Because the tracheal hole was compressed by the myocutaneous flap, a cannula had to be inserted into the tracheal hole to prevent obstruction after surgery. Hence, a pressure ulcer formed because of a decrease in blood flow at the site where the major pleurocutaneous flap was sutured and the region of the tracheostomy that was posterior to the cannula, and this was thought to be the cause of the re-fistula.

Therefore, during our second closure procedure, we overcame the working space problem by using HFJV intraoperatively. By avoiding the use of the operated neck skin and using the paratracheal and foramen skin of the pectoralis major flap that was elevated during the first procedure, we elevated a local hinged flap to increase the reliability of the blood flow to the flap. We then planted an ultra-thin skin graft on the back of the hinged flap according to the method previously described by Guo et al. so that the skin graft was prelaminated and the hinged flap was delayed at the same time [16,17]. This improved the reliability of the blood flow to the flap, reduced the size of the pectoralis major flap, increased the space around the tracheostomy, and reduced pressure due to the flap on the tracheal hole, thereby eliminating the need to insert a cannula during postoperative respiratory management.

HFJV is a method of respiratory management that uses high-frequency jet ventilation from tracheal tubes with very thin 14- to 16-gauge cuffless tubes and is used during surgery in patients in whom positive-pressure ventilation was not possible, such as those with tracheal injury or bronchial fistula or those who are at a high risk of ventilator injury [18]. In this case, the intubation tube was omitted by selecting HFJV for respiratory management to maximize the working space intraoperatively. Problems associated with HFJV include ease of CO2 accumulation and the need for frequent blood gas analysis because of the inability to monitor CO2 in the exhaled breath. It is also especially important to communicate with the anesthesiologists intraoperatively. HFJV can be used to provide respiratory support in patients other than those with permanent tracheal holes. Accordingly, it may be useful in the field of plastic surgery in cases where facial adjustment is required because it reduces deformation of the angle of the mouth due to the intubation tube and blockage of the operative field by the perifacial anesthesia circuit. However, the prevalence of severe acute respiratory syndrome coronavirus 2 infection requires caution in order to prevent the infection from spreading.

## Conclusions

We encountered a case where an intractable TE fistula occurred due to a TE shunt after the patient underwent total pharyngolaryngeal resection for hypopharyngeal cancer and reconstruction with a flap of free jejunum.

Once the esophagus is reconstructed after pharyngeal esophagectomy, surgical closure interventions, such as myocutaneous flaps, are often required. However, the size of the flap may be limited by the location of the operative visual field and trachea, thus the choice of flap size must be carefully studied in each case. HFJV is useful as it ensures an adequate working space in the narrow surgical field of the neck.
